# Presurgical molecular therapy for renal cell carcinoma with venous tumor thrombus: a systematic review and meta-analysis

**DOI:** 10.3389/fimmu.2025.1705494

**Published:** 2025-11-28

**Authors:** Kewei Chen, Lin Zhuo, Zhuo Liu, Liyuan Ge, Le Yu, Shudong Zhang

**Affiliations:** 1 Department of Urology, Peking University Third Hospital, Beijing, China; 2 Key Laboratory of Epidemiology of Major Diseases (Peking University), Ministry of Education

**Keywords:** renal cell carcinoma, tumor thrombus, presurgical molecular therapy, radical nephrectomy and thrombectomy, tyrosine kinase inhibitors

## Abstract

**Background:**

Presurgical molecular therapy (PMT) including tyrosine kinase inhibitors (TKIs) and immune checkpoint inhibitors (ICIs) showed various outcomes for renal cell carcinoma (RCC) with tumor thrombus (TT). We aimed to evaluate the impact of PMT on Mayo level or TT height and the treatment-related adverse events (AEs).

**Methods:**

A systematic literature search was conducted in PubMed, Embase, Cochrane Library, and Web of Science up to June 2023 to identify relevant studies investigating the impact of PMT on RCC patients with TT. The literature investigating the impact of PMT on RCC patients with venous TT, whether followed by surgery or not, was included.

**Results:**

Overall, 184 patients were enrolled in this study. 30.7% (95% CI, 17.6–43.8%, I^2^ = 79%, p<0.01) patients experienced a decrease in TT levels after receiving PMT, while only 1.5% (95% CI, 0–0.044%, I^2^ = 0%, p=0.98) exhibited an increase in TT levels. An average decrease of 15.2mm (95% CI, 22.4–8.0, I^2^ = 77%, p<0.01) of TT in 117 patients was observed after PMT. The most common AEs was hypertension (49.9%, 95% CI, 27.1–77.7, I^2^ = 88%, p<0.01), diarrhea (20.2%, 95% CI, 2.7–37.6, I^2^ = 83%, p<0.01), fatigue (25.3%, 95% CI, 6.1–44.4, I^2^ = 84%, p<0.01) and hand-foot syndrome (25.5%, 95% CI, 5.6–45.5, I^2^ = 86%, p<0.01).

**Conclusion:**

PMT is available to assist in lowering the TT level in RCC patients aiming to simply the surgical procedures, particularly in patients with Mayo grade 3/4. The frequency and severity of AEs during PMT are tolerable.

**Systematic Review Registration:**

https://www.crd.york.ac.uk/prospero/, identifier CRD420234399128.

## Introduction

Renal cell carcinoma (RCC) constitutes approximately 3% of all cancers, ranking third among urinary system tumors (1, 2). RCC exhibits a distinct biologic propensity for vascular invasion, with 4-15% of cases developing renal vein or inferior vena cava (IVC) tumor thrombus (TT), and 30-50% of these patients experience distant metastasis (3–5). Radical nephrectomy and thrombectomy (RNAT) remains the standard treatment for RCC with TT, improving the prognosis to 40%-65% in 5-year cancer-specific survival (6). Nevertheless, the surgical procedures, particularly TT resection and IVC reconstruction, carry a high risk of surgical morbidity and mortality and its rates escalate with increasing TT level (7–10). Therefore, reducing TT level to simplify surgical procedures and mitigate perioperative risk represent a critical clinical need (11, 12). Presurgical molecular therapy (PMT) including tyrosine kinase inhibitors (TKIs) and immune checkpoint inhibitors (ICIs) has shown the potential to reduce TT level, making it possible to reduce surgical difficulty. Cost et al. conducted the initial retrospective study on PMT for RCC with TT and observed that sunitinib positively impacts TT regression (13). However, the clinical significance and relevance remain unclear. Subsequent investigations suffer from limitations including small sample sizes, single-center designs, and heterogeneous populations, leading to divergent conclusions, rendering this a contentious clinical issue (14–17). Presently, the inaugural prospective study, NAXIVA, demonstrates that 37.5% patients experienced a decrease in TT grade, and 75% exhibited a reduction in TT height following standardized axitinib therapy (18). Nevertheless, there is still a lack of large-scale clinical studies to verify the efficacy and safety of PMT. Therefore, given the mixed early evidence for PMT, a systematic synthesis of existing data is urgently needed to clarify the impact of PMT on TT downstaging, as well as its safety profile. This study conducted a systematic review and meta-analysis to comprehensively evaluate the effect of PMT on Mayo level and height of TT in RCC patients, with the aim to provide evidence to guide clinical decision-making for this high-risk cohort.

## Methods

This study was conducted in accordance with the Preferred Reporting Items for Systematic Reviews and Meta-analysis (PRISMA) criteria. The review protocol for this study was registered on PROSPERO (CRD420234399128).

### Search strategy

We conducted a systematic literature search in PubMed, Embase, Cochrane Library, and Web of Science up to June 2023 to identify relevant studies investigating the impact of PMT on RCC patients with TT. To ensure the transparency and rigor of the study design in line with the PRISMA guidelines, the systematic review and meta-analysis was structured by the PICO (Population, Intervention, Comparison, Outcome) framework. Population (P): Patients with pathologically confirmed RCC with TT. Intervention (I): PMT as the experimental intervention, including TKIs (e.g., sunitinib, sorafenib, axitinib, pazopanib), ICIs (e.g., nivolumab, ipilimumab, pembrolizumab, avelumab) or both. PMT was administered preoperatively, with regimens (drug type, dosage, and treatment cycle) clearly documented in the included studies. Comparison (C): Given the scarcity of standard treatment of PMT for RCC with TT, the present analysis focused on the intra-intervention effect of PMT, namely changes in TT indicators before and after PMT rather than a direct head-to-head comparison. This approach aligns with the core objective of evaluating whether PMT can alter TT status, which is consistent with the exploratory nature of current research in this field. Outcome (O): The primary outcomes include Mayo grade and TT height, which is based on their clinical relevance to surgical management and prognosis of RCC with TT. Mayo grade directly determines surgical complexity and perioperative risk. The key metrics for this outcome included the proportion of patients with TT grade downstaging and grade upstaging. TT height is a continuous outcome measured in millimeters (mm), representing the maximum longitudinal length of TT in the venous system (assessed via imaging before and after PMT). Decrease of TT height can simplify surgical dissection and reduce the need for complex IVC reconstruction, thereby lowering perioperative morbidity. The key metric for this outcome was the average change in TT height before and after PMT. Secondary Outcome: Treatment-related adverse events (AEs) of PMT to assess the safety of PMT.

Separate searches were performed using population ((renal cell carcinoma, renal cell cancer, renal tumor, kidney cancer, kidney carcinoma, renal neoplasm, kidney neoplasm) and (tumor thrombus, tumor thrombosis, tumor embolus, inferior vena cava thrombus, venous tumor thrombus, venous thrombus)), intervention (neoadjuvant therapy, presurgical therapy, target molecular therapy, tyrosine kinase inhibitor, immunotherapy, sunitinib, sorafenib or pazopanib, axitinib, cabozantinib, temsirolimus, lapatinib, pembrolizumab, nivolumab, Ipilimumab, bevacizumab). Furthermore, we examined the identified original papers, reviews, meta-analyses, and comments that were included in the references from the pertinent research.

### Inclusion criteria and study eligibility

The present study enrolled patients diagnosed with RCC and venous TT who underwent PMT involving TKIs, ICIs, or both. The inclusion criteria encompassed literature investigating the impact of PMT on RCC patients with venous TT, whether followed by surgery or not. Exclusion criteria comprised the following: (1) fundamental research studies; (2) studies only concentrating on RCC without TT; (3) non-original articles (such as reviews, editorials, comments, letters, editorials, systematic reviews, and meta-analysis); (4) gray literature (e.g., thesis, abstracts only); (5) studies lacking data on TT changes after PMT. In order to maintain the homogeneity of the cases included in the study, we strictly screened the literature according to the following inclusion criteria: (1) studies must explicitly confirm RCC diagnosis via pathological examination; (2) venous TT must be verified by imaging modalities including computed tomography, magnetic resonance imaging, or contrast-enhanced ultrasound; (3) drug type, dosage, and treatment cycle of PMT must be clearly documented; (4) post-PMT changes in TT (Mayo grade or height) must be available for assessment. In cases where multiple studies examined the same variable at the same endpoint, the data were merged. The most illuminating study was chosen with the biggest sample size when different studies within the same patient cohort reported the same characteristic. Two authors (K.C. and L.Z.) independently reviewed titles and abstracts, resolving any disagreements through discussion with senior authors (Z.L).

### Quality assessment

The quality of the included prospective studies was assessed using the Methodological Evaluation Metrics for Non-Randomized Controlled Trials (MINORS) (19). Twelve assessment indications make up MINORS, and each one has a score range of 0 to 2. 0 indicates that there is no data reported. 1 indicates that there is data reported, but not enough details. 2 indicates that there is enough information in the data report. The retrospective studies without comparison group were assessed by JBI Critical Appraisal Checklist for Case Series (20).

### Data extraction

The selection of studies was carried out independently by two investigators (K.C. and L.Z.), and any discrepancies between the two would be discussed jointly by the third author (Z.L.). The following details were noted about the characteristics of the included studies: authors, year, study design, country, sample size, therapeutic drug, therapeutic period, number of patients, age, reported endpoints, and AEs. While original data were hardly accessed, the data was extracted from the histogram or line chart by software Engauge Digitizer version 10.8.

### Statistical analysis

The statistical methodology employed in this study involved the random-effect model or the fixed-effect model after double arcsine conversion. The effect size for all combined results was expressed using 95% confidence intervals (CI) with upper and lower limits. To assess statistical heterogeneity, we utilized both the Cochrane Q statistic and the I^2^ statistic. Specifically, if the p-value from the Cochran Q test was less than 0.05 or the I^2^ statistic exceeded 50%, significant heterogeneity among the literature was present, and we employed a random-effect model. Otherwise, a fixed-effect model was used. Additionally, we conducted sensitivity analysis by systematically excluding each individual study to evaluate the stability of our findings. All statistical analyses were conducted by R software (version 3.2.2, Mac).

## Results

### Study selection and characteristics

**Figure 1:**
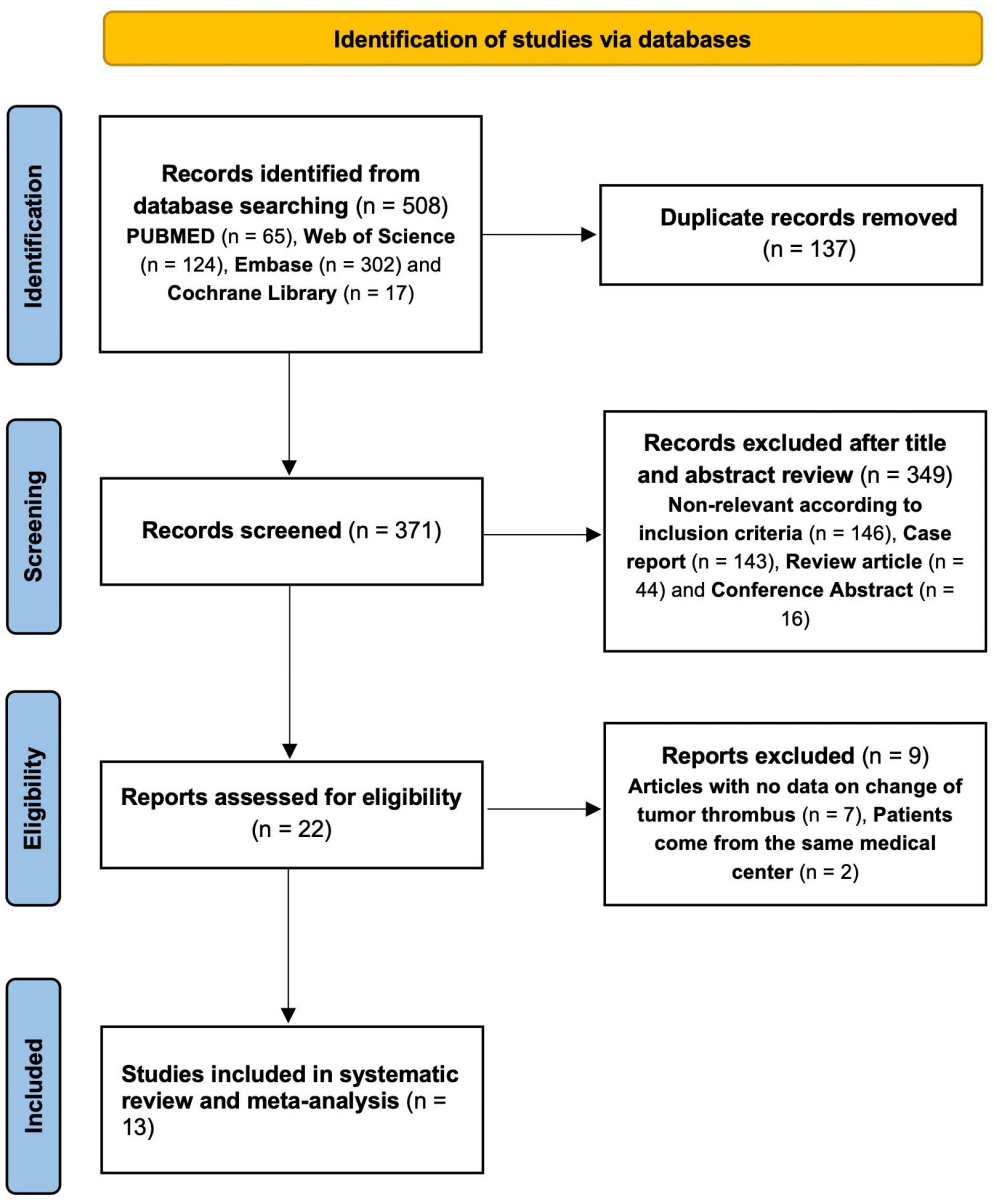
Flow chart of the selection of publications included in the meta-analysis.

The initial search yielded 508 relevant references in PubMed (n=65), Web of science (n=124), Embase (n=302) and the Cochrane Library (n=17). After removing duplicate studies and conducting thorough screenings of titles, abstracts, and full-texts, 13 studies were ultimately included in our analysis. These comprised 1 prospective study (18) and 12 retrospective studies (13, 14, 17, 21–29), involving a total of 184 patients. Detailed study characteristics are presented in **Table 1**. Among the 13 studies, 11 focused on targeted therapy and 2 investigated a combination of targeted therapy and immunotherapy. The study selection process is shown in **Figure 1**.

**Table 1:** Characteristic of included studies.

First author	Year	Study design	Country	N	Drug	Duration	Subtype	Adverse events
Nicholas G. Cost (13)	2011	Retrospective	America	25	Sunitinib (n=12) Bevacizumab (n=9) Temsirolimus (n=3) Sorafenib (n=1)	Sunitinib (3 cycles, range 2-6) Bevacizumab, Temsirolimus or Sorafenib (2 cycles, range 1-3)	19 clear cell; 3 unclassified	NR
Pierre Bigot (28)	2014	Retrospective	France	14	Sunitinib (n=11) Sorafenib (n=3)	Sunitinib (2 cycles, range 2-5) Sorafenib (2 cycles, range 2-3)	14 clear cell	NR
Yushi Zhang (21)	2015	Retrospective	China	5	Sorafenib (n=5)	Sorafenib (96 days, range 30–278 days)	5 clear cell	NR
Takeshi Ujike (25)	2016	Retrospective	Japan	7	Sunitinib (n=7)	Sunitinib (1 cycles, range 1-4)	2 clear cell; 5 unknown	NR
Hironori Fukuda (14)	2017	Retrospective	Japan	21	Sunitinib (n=17) Sorafenib (n=1) Pazopanib (n=1) Temsirolimus (n=1)	Sunitinib, Sorafenib, Pazopanib, Temsirolimus (3 months, range 0.8–21 months)	10 clear cell; 3 papillary; 2 Sarcomatoid change; 6 unknown	NR
Yoshimi Tanaka (24)	2017	Retrospective	Japan	10	Axitnib (n=10)	Axitnib (3.9 months, range 3.1-6.1)	10 clear cell	Yes
Cheng Peng (29)	2018	Retrospective	China	18	Sunitinib (n=9) Sorafenib (n=6) Axitinib (n=3)	Sunitinib, Sorafenib, Axitinib (2 cycles, range 1-3)	15 clear cell; 2 papillary; 1 chromophobe	Yes
Wen Cai (27)	2018	Retrospective	China	23	Sunitinib (n=14) Sorafenib (n=9)	Sunitinib, Sorafenib (2.5 months)	19 clear cell; 4 papillary	Yes
Yasuyoshi Okamura (17)	2019	Retrospective	Japan	9	Pazopanib (n=9)	Pazopanib (3 months)	7 clear cell; 1 clear cell with sarcomatoid; 1 unknown	Yes
Charles A. Field (26)	2019	Retrospective	America	19	Sunitinib (n=19)	Sunitinib (3 cycles, range 2-5)	19 clear cell	NR
Grant D. Stewart (18)	2022	Prospective	Britain	20	Axitnib (n=20)	Axitnib (2 months)	20 clear cell	Yes
Kazuhiko Yoshida (23)	2022	Retrospective	Japan	5	Nivolumab and ipilimumab (n=3), Pembrolizumab and axitinib (n=2)	Nivolumab and ipilimumab (18 months, range 8-20), Pembrolizumab and axitinib (2 months, range 1-3)	4 clear cell; 1 unknown	Yes
Taisuke Tobe (22)	2023	Retrospective	Japan	6	Avelumab and axitinib (n=6)	Avelumab and axitinib (3 months)	5 clear cell; 1 unknown	Yes

### Quality assessment

The quality assessment results of all included articles are acceptable for the present meta-analysis (**Table 2**).

**Table 2:** Quality assessment of included studies.

A. JBI critical appraisal checklist for case series for included retrospective studies											
Study	Q1	Q2	Q3	Q4	Q5	Q6	Q7	Q8	Q9	Q10	Overall appraisal
Nicholas G. Cost 2011 (13)	Yes	Yes	Yes	Yes	Yes	Yes	Yes	Yes	Yes	Yes	Include
Pierre Bigot 2014 (28)	Yes	Yes	Yes	Yes	Yes	Yes	Yes	Yes	No	Yes	Include
Yushi Zhang 2015 (21)	Yes	Yes	Yes	Yes	Yes	Yes	Yes	Yes	Yes	Yes	Include
Takeshi Ujike 2016 (25)	Yes	Yes	Yes	Yes	Yes	Yes	Yes	Yes	No	Yes	Include
Hironori Fukuda 2017 (14)	Yes	Yes	Yes	Yes	Yes	Yes	Yes	Yes	Yes	Yes	Include
Yoshimi Tanaka (24)	2017	Yes	Yes	Yes	Yes	Yes	Yes	Yes	Yes	Yes	Include
Cheng Peng 2018 (29)	Yes	Yes	Yes	Yes	Yes	Yes	Yes	Yes	Yes	Yes	Include
Wen Cai 2018 (27)	Yes	Yes	Yes	Yes	Yes	Yes	Yes	Yes	No	Yes	Include
Yasuyoshi Okamura 2019 (17)	Yes	Yes	Yes	Yes	Yes	Yes	Yes	Yes	No	Yes	Include
Charles A. Field 2019 (26)	Yes	Yes	Yes	Yes	Yes	Yes	Yes	Yes	No	Yes	Include
Kazuhiko Yoshida 2022 (23)	Yes	Yes	Yes	Yes	Yes	Yes	Yes	No	No	Yes	Include
Taisuke Tobe 2023 (22)	Yes	Yes	Yes	Yes	Yes	No	Yes	No	No	Yes	Include

B. MINORS index for included non-randomized studies.											
Study	I	II	III	IV	V	VI	VII	VIII	Total		
Grant D. Stewart 2022 (18)	2	2	2	2	2	2	2	2	16		

Note: numbers Q1-Q10 in heading signified: Q1, were there clear criteria for inclusion in the case series? Q2, was the condition measured in a standard, reliable way for all participants included in the case series? Q3, were valid methods used for identification of the condition for all participants included in the case series? Q4, did the case series have consecutive inclusion of participants? Q5, did the case series have complete inclusion of participants? Q6, was there clear reporting of the demographics of the participants in the study? Q7, was there clear reporting of clinical information of the participants? Q8, were the outcomes or follow up results of cases clearly reported? Q9, was there clear reporting of the presenting site(s)/clinic(s) demographic information? Q10, was statistical analysis appropriate?

Note: numbers I-VIII in heading signified: I, a clearly stated aim; II, inclusion of consecutive patients; III, prospective collection of data; IV, endpoints appropriate to the aim of the study; V, unbiased assessment of the study endpoint; VI, follow-up period appropriate to the aim of the study; VII, loss of follow up less than 5%; VIII, prospective calculation of the study size.

### Tumor thrombus response

**Figure 2:**
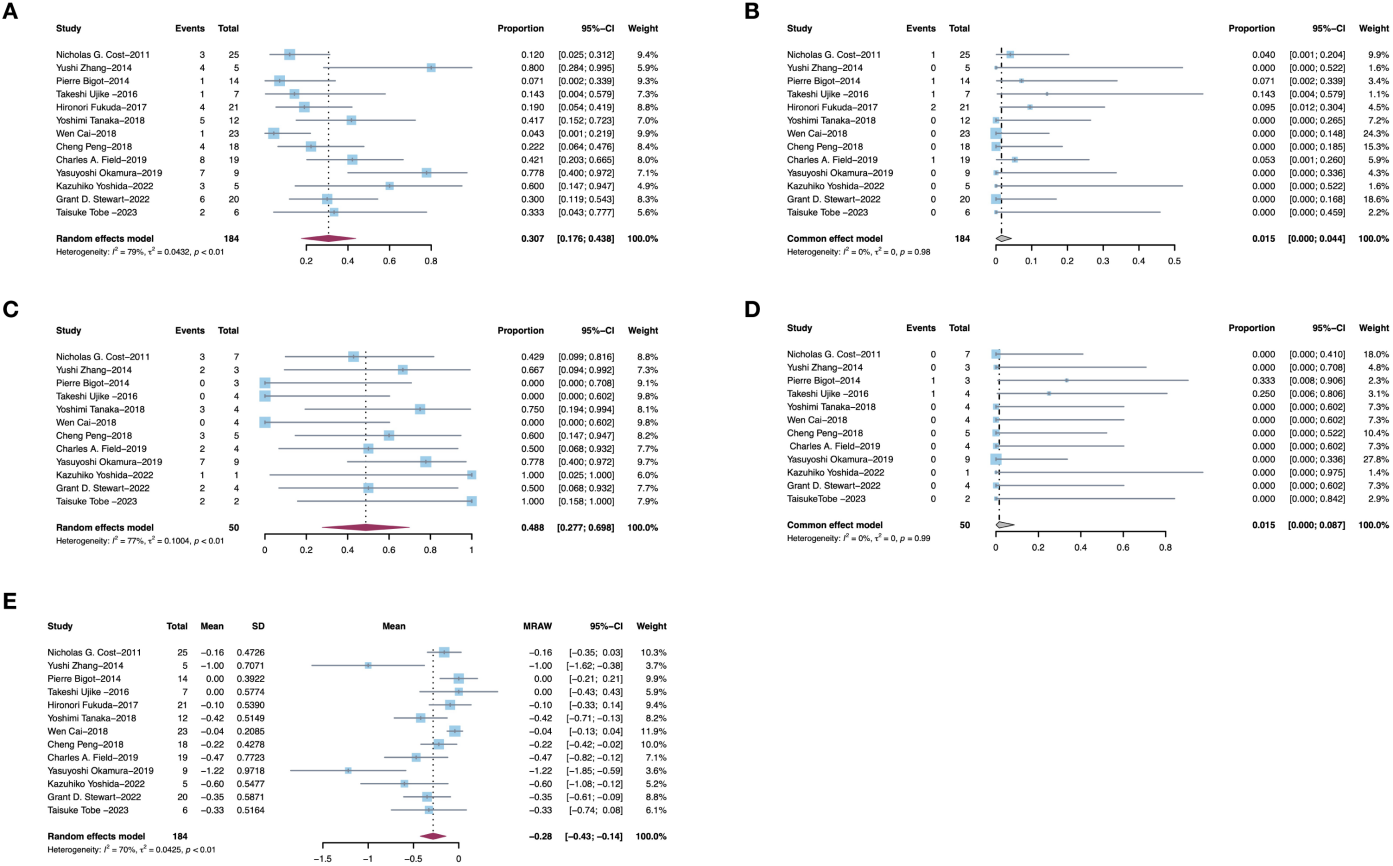
Pooled estimates of efficacies of presurgical molecular therapy on Mayo grade for renal cell carcinoma with tumor thrombus. **(A)** Downstage Mayo grade. **(B)** Upstage Mayo grade. **(C)** Downstage Mayo grade 3/4. **(D)** Upstage Mayo grade 3/4. **(E)** Average Mayo grade changes.

**Figure 3:**
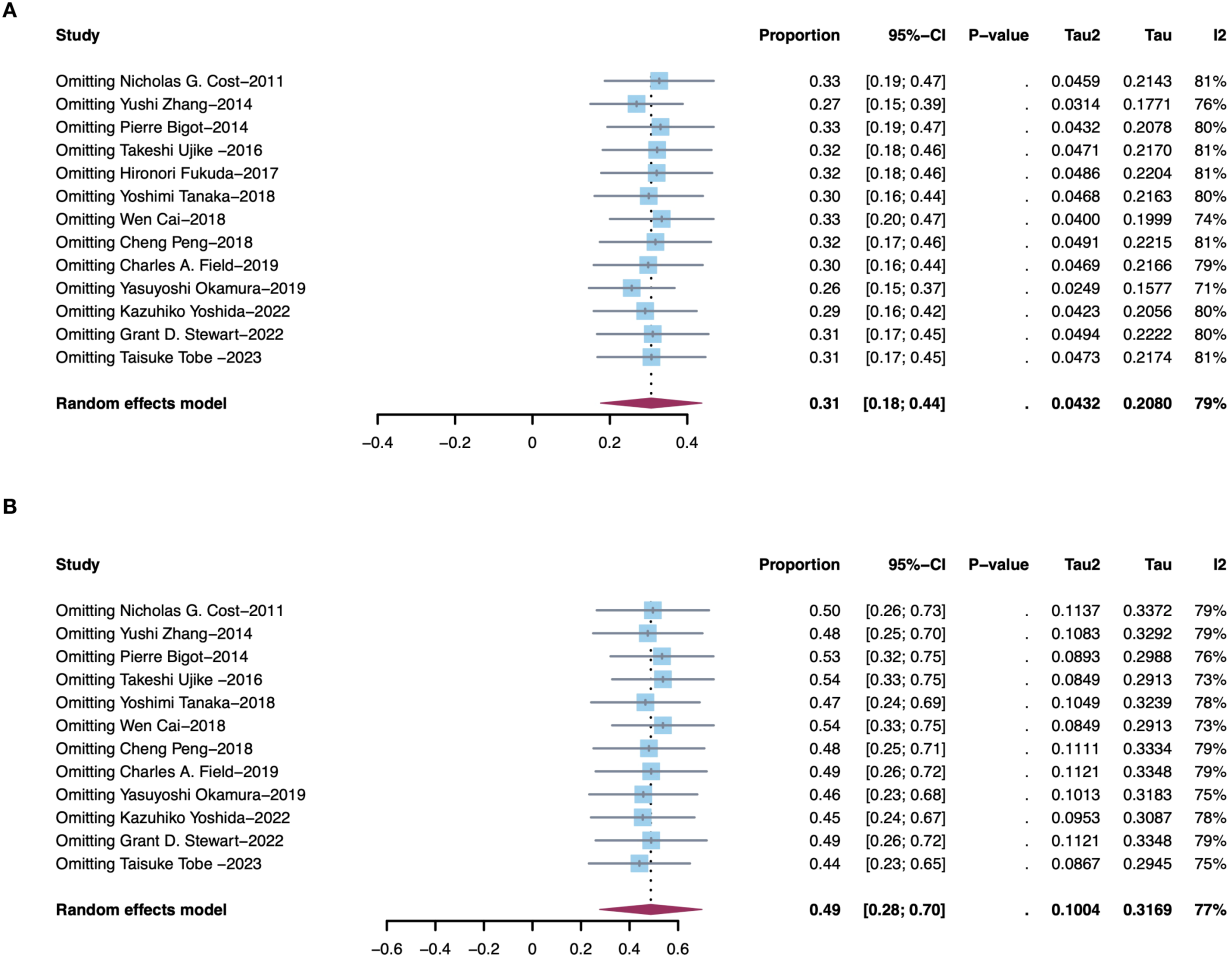
Sensitivity analysis of reduced tumor thrombus levels. **(A)** Downstage Mayo grade of all patients. **(B)** Downstage Mayo grade of patients with Mayo grade 3/4.

Thirteen studies provided data of TT levels in RCC patients after receiving PMT. Among 184 patients, 30.7% (95% CI, 17.6–43.8%, I^2^ = 79%, p<0.01, **Figure 2A**) patients experienced a decrease in TT levels after receiving PMT, while only 1.5% (95% CI, 0–0.044%, I^2^ = 0%, p=0.98, **Figure 2B**) exhibited an increase in TT levels. Additionally, we specifically analyzed patients with Mayo 3/4 TT. Among these patients, 48.8% (95% CI, 27.7%–69.8%, I^2^ = 77%, p<0.01, **Figure 2C**) experienced a decrease in TT level, while only 1.5% (95% CI, 0–8.7%, I^2^ = 0%, p=0.99, **Figure 2D**) exhibited an increase. Further analysis of the changes in TT levels in the above studies showed that the average TT level decreased by 0.28 grade (95% CI, 0–0.044%, I^2^ = 0%, p=0.98) (**Figure 2E**). Sensitivity analysis of reduced tumor thrombus levels is shown in **Figure 3** and other sensitivity analysis about TT level changes were shown in **Supplementary Figure 1**.

**Figure 4:**
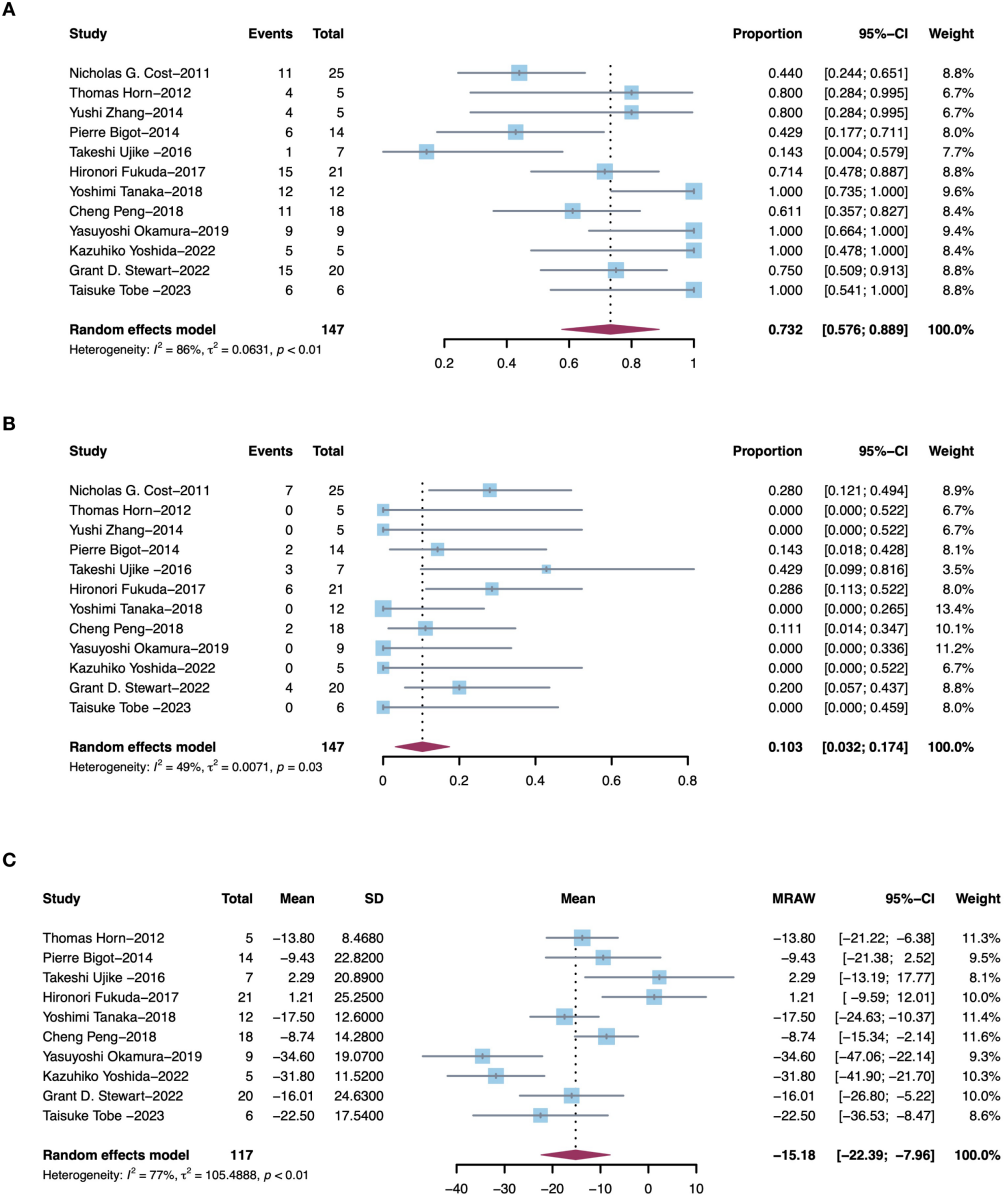
Pooled estimates of efficacies of presurgical molecular therapy on thrombus height for renal cell carcinoma with tumor thrombus. **(A)** Decrease in thrombus height. **(B)** Increase in thrombus height. **(C)** Average thrombus height changes.

12 studies simultaneously assessed changes in TT height following PMT. Among these patients, 73.2% (95% CI, 57.6%–88.9%, I^2^ = 86%, p<0.01, **Figure 4A**) experienced a decrease in TT height and 10.3% (95% CI, 3.2%–17.4%, I^2^ = 86%, p<0.01, **Figure 4B**) experienced an increase in TT height. Among the above studies, 10 reported absolute height changes of TT, with an average decrease of 15.2mm (95% CI, 22.4–8.0, I^2^ = 77%, p<0.01, **Figure 4C**) in 117 patients after PMT. Sensitivity analysis about TT height changes were shown in **Supplementary Figure 2**.

### Adverse events

**Figure 5:**
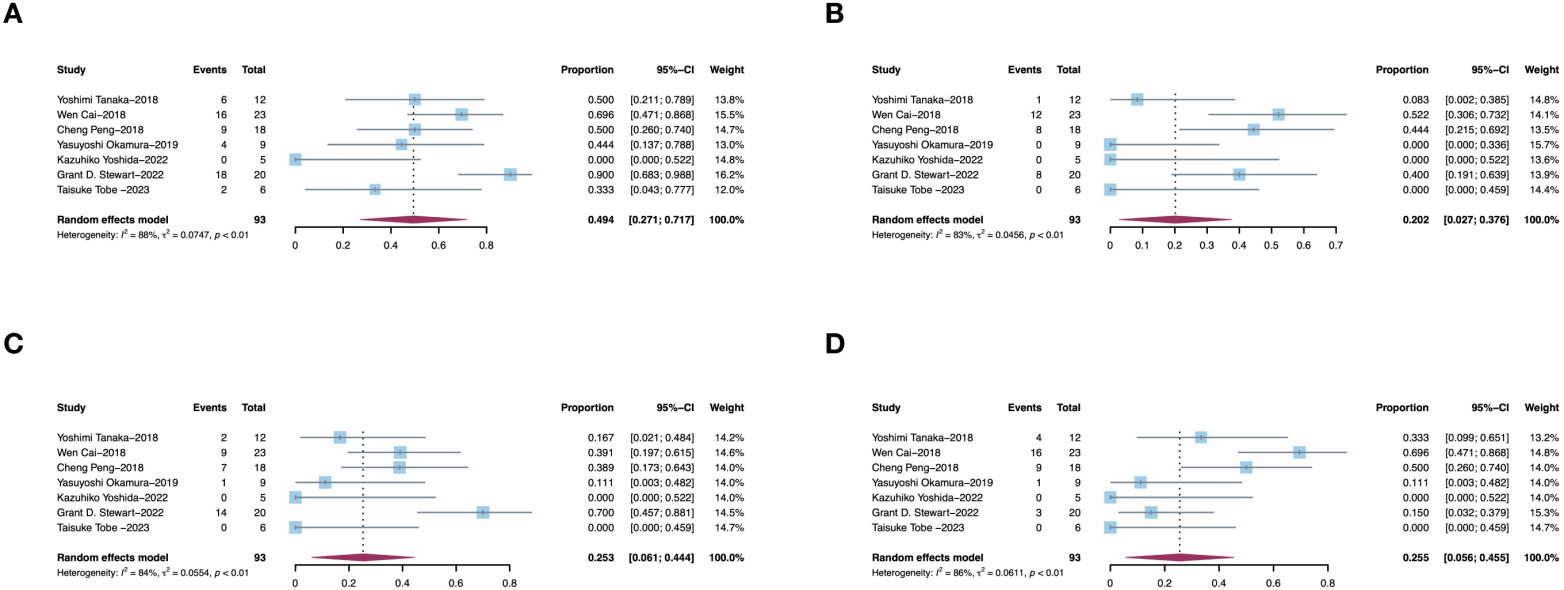
Pooled estimates of adverse effects of presurgical molecular therapy on for renal cell carcinoma with tumor thrombus. **(A)** hypertension. **(B)** diarrhea. **(C)** fatigue. **(D)** hand-foot syndrome.

AEs reported in the included literature include hypertension, hypothyroidism, fatigue, hand-foot syndrome, diarrhea, nausea, leucopenia, anemia, thrombocytopenia, liver function abnormalities, mucositis and anorexia. The most common AEs was hypertension (49.9%, 95% CI, 27.1–77.7, I^2^ = 88%, p<0.01, **Figure 5A**), diarrhea (20.2%, 95% CI, 2.7–37.6, I^2^ = 83%, p<0.01, **Figure 5B**), fatigue (25.3%, 95% CI, 6.1–44.4, I^2^ = 84%, p<0.01, **Figure 5C**) and hand-foot syndrome (25.5%, 95% CI, 5.6–45.5, I^2^ = 86%, p<0.01, **Figure 5D**). Sensitivity analysis about AEs were shown in **Supplementary Figurel 3**.

## Discussion

A well-documented characteristic of RCC is its propensity for vascular invasion, which manifests as TT in the renal vein or IVC in 4-15% of cases (2, 6). In cases of non-metastatic RCC with vascular invasion, RNAT represents the standard treatment, which significantly raises the 5-year cancer-specific survival rate (30, 31). However, the considerable technical complexity of RNAT, particularly for high-level TT, especially Mayo 3/4 TT, carries a substantial risk of perioperative morbidity and mortality (32–34). The complication rate demonstrated considerable variation depending on the TT level, with reported ranges of 12.4% to 46.9% (9, 34). Especially, the morbidity rate escalates with the level of TT, reaching 10-40% in Mayo 4 patients (35). Consequently, PMT to reduce TT level and facilitate less complex surgery has emerged as a potential strategy (36–38). The first prospective study, NAXIVA, evaluated the efficacy of neoadjuvant therapy in RCC with TT, which demonstrated that PMT effectively reduced TT levels, thereby facilitating surgical complexity (18, 39). Furthermore, several retrospective studies have explored the feasibility of PMT in RCC with TT, however, their conclusions exhibit substantial heterogeneity, primarily attributable to the study design (23, 26, 29, 39). Consequently, the synthesis of current study data to confirm the function of PMT in RCC with TT has vital clinical value, which provides preliminary evidence for subsequent clinical studies.

The efficacy of PMT in RCC with TT is subject to be controversial, as evidenced by heterogeneous study outcomes (15, 16, 40). Overall, retrospective investigations have produced inconsistent findings, with TT level reductions reported in 7.1% to 41.7% of patients (26, 28, 38). Cost et al. documented a 44% decline in TT height and a 48% reduction in primary lesions. However, the median TT height decrease was below 1 cm, and only 12% of patients achieved the outcome of lowering TT level (13). Bigot et al. further illustrated this variability, with merely 1 of 14 patients showing a TT reduction from Mayo 2 to 1, while one patient experienced TT escalation from Mayo 3 to 4, impeding surgical resection (28). Conversely, more optimistic data emerge from other studies. Karakiewicz et al. initially reported sunitinib induced TT reduction that simplified surgery and more case reports demonstrated consistent results (39). In addition, more case series studies have shown that PMT can effectively reduce TT levels and simplify surgical procedures. Peng et al. reinforced these positive trends, observing significant TT size decreases in 61.1% of patients, including 60% of those with Mayo 3/4 TT (29). Supporting this, the prospective study, NAXIVA, demonstrated marked TT height reductions with preoperative axitinib, resulting in altered surgical approaches for 41.1% of cases (18). As ICIs are gradually applied to RCC, some clinical studies have reported more positive results. Studies based on ICIs have demonstrated a decrease in TT level in 33.3% to 60% of patients, suggesting that future PMT strategies incorporating ICIs may represent a more promising therapeutic approach (23).

Through a systematic review, we executed a comprehensive meta-analysis aiming to derive new insights into molecular therapeutics for RCC with TT. This analysis included 13 single-arm studies comprising 184 patients. Of the 184 participants, 30.7% demonstrated a decrease in TT Mayo level after PMT, with a mean reduction of 0.28 in TT grade. These findings provide preliminary evidence for the feasibility of PMT in reducing TT levels and facilitating surgical procedures. Owing to substantial heterogeneity across studies, sensitivity analysis was applied to test the robustness of conclusions. In sensitivity analysis, after successive exclusion, the fluctuation range of the combined effect of TT reduction ratio is 26%-33%. It is important to note that the application of PMT has been constrained by the highly invasive nature of TT and TKIs or ICIs might not effectively restrain tumor progression (41–43). Nevertheless, our results showed that merely 1.5% of patients had elevated TT levels during therapy. Therefore, the principal apprehension that TT progression under PMT could exacerbate surgical challenges and worsen outcomes is mitigated. Subgroup analysis concentrated on RCC patients with Mayo 3/4 TT, who face elevated perioperative risks (30, 44–46). In this subgroup, 48.8% exhibited a decrease in TT level, compared to only 1.5% with an increase, indicating that PMT is particularly applicable to cases with high Mayo level (32, 47–49). Furthermore, as surgical complexity correlates not only with Mayo level but also TT height, we analyzed this outcome separately. Overall, 73.2% of analyzed patients had a reduction in TT height, versus 10.3% with an increase. Additional analyses revealed an average TT height decrease of 15.2 mm after PMT. Therefore, these data preliminarily demonstrate the feasibility of PMT in reducing TT levels.

The management of AEs is also very important during PMT (50–52), so this outcome is also analyzed. The results showed that the common AEs were hypertension, diarrhea, fatigue, and hand-foot syndrome. Although the incidence of hypertension is high, most patients experience relief after drug treatment. The incidence of AEs including diarrhea, fatigue, hand-foot syndrome and other AEs is relatively low, and very few patients stop treatment due to serious AEs. This helps to improve the application of PMT in RCC patients with TT (53, 54).

In addition, with the exploration of ICIs, therapy based on ICIs for preoperative intervention seems feasible, however, although some studies have reported the effect of combination therapy, the current level of evidence is insufficient due to its small sample size (23, 50). In this study, the response rate of PMT based on ICIs was higher than that based on TKIs. Despite the potential for a greater reduction rate among ICIs treated patients relative to targeted therapy (44.9% vs 29.1%), the small case numbers restrict the generalizability of this result. However, it suggests that ICIs could potentially benefit more patients in this field. Consequently, more extensive clinical research is needed to verify the effectiveness and safety of ICIs.

This study provides preliminary evidence for PMT in RCC patients with TT to reduce the difficulty of surgery, but there are some limitations. First of all, most of the literatures included in this study were retrospective studies with small individual sample sizes, and the total sample size of the meta-analysis (184 patients) remains relatively small. This is mainly attributed to the low incidence of venous TT in RC, resulting in a limited number of eligible cases in clinical practice. Secondly, the present analysis combined data from patients receiving TKIs, ICIs, or combinations, considering that the mechanisms of TKIs and ICIs are not the same, which may obscure potential differences in efficacy and safety between different PMT regimens. At the same time, the effects of different drugs are not compared, because there is no consensus on the current PMT for such patients. Finally, due to the absence of follow-up results from most studies, it is unclear whether PMT improves outcomes. Future research should prioritize multicenter, prospective studies with larger sample sizes to verify the efficacy and safety of PMT in RCC patients with venous TT. As more clinical data accumulate (especially data from phase II/III clinical trials), updated meta-analyses with expanded sample sizes can be conducted to further validate or refine the conclusions of this preliminary study.

## Conclusion

PMT is available to assist in lowering the TT level in RCC patients through exploring the literature and analyzing data from the preceding 20 years, particularly in patients with Mayo grade 3/4. This conclusion is a preliminary extrapolation given the presence of selection bias and other confounding factors in the included literature, which gives the base to design a prospective study. Further conclusions would be facilitated by including more clinical studies with large scale and conducting an exploration of drug categories.

## Data Availability

The original contributions presented in the study are included in the article. Further inquiries can be directed to the corresponding author.

## Supplementary material

Supplementary FIGURE 1 Sensitivity analysis of reduced tumor thrombus levels. **(A)** Upstage Mayo grade. **(B)** Upstage Mayo grade 3/4. **(C)** Average Mayo grade changes.

Supplementary FIGURE 2 Sensitivity analysis of changes of tumor thrombus. **(A)** Decrease in thrombus height. **(B)** Increase in thrombus height. **(C)** Average thrombus height changes.

Supplementary FIGURE 3 Sensitivity analysis of adverse effects. **(A)** hypertension. **(B)** diarrhea. **(C)** fatigue. **(D)** hand-foot syndrome.
