# Supplementary figures and images for: Presurgical molecular therapy for renal cell carcinoma with venous tumor thrombus: a systematic review and meta-analysis
Source: Front Immunol. 2025 Nov 28;16:1705494. doi: 10.3389/fimmu.2025.1705494 (PMC12698656; doi:10.3389/fimmu.2025.1705494)

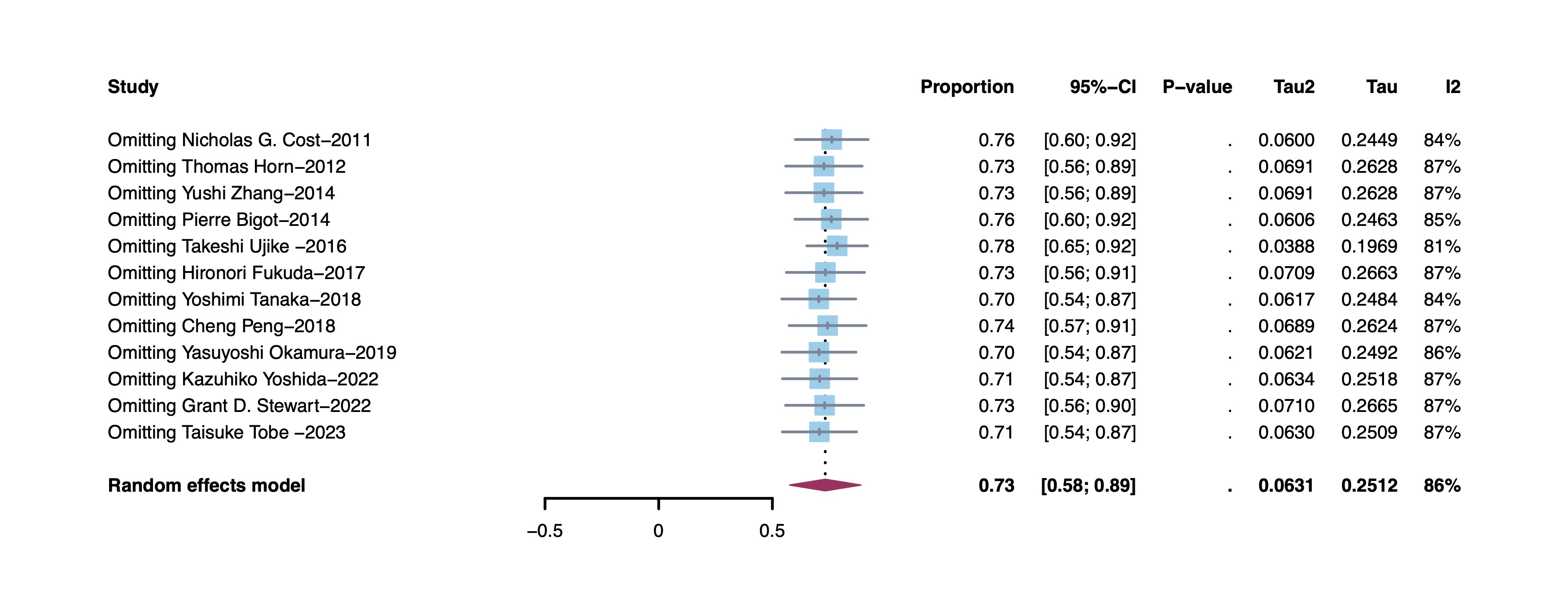

Supplement: Supplementary file 1 [file Image4.jpeg]

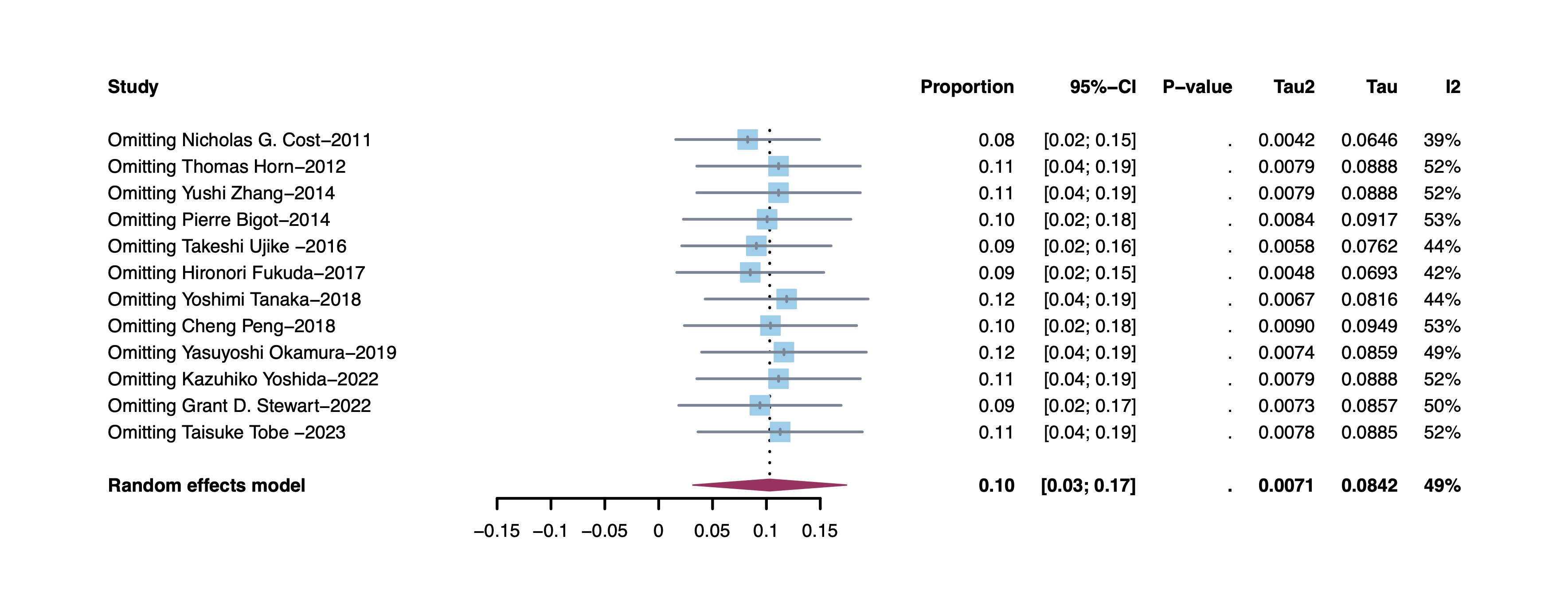

Supplement: Supplementary file 2 [file Image5.jpeg]

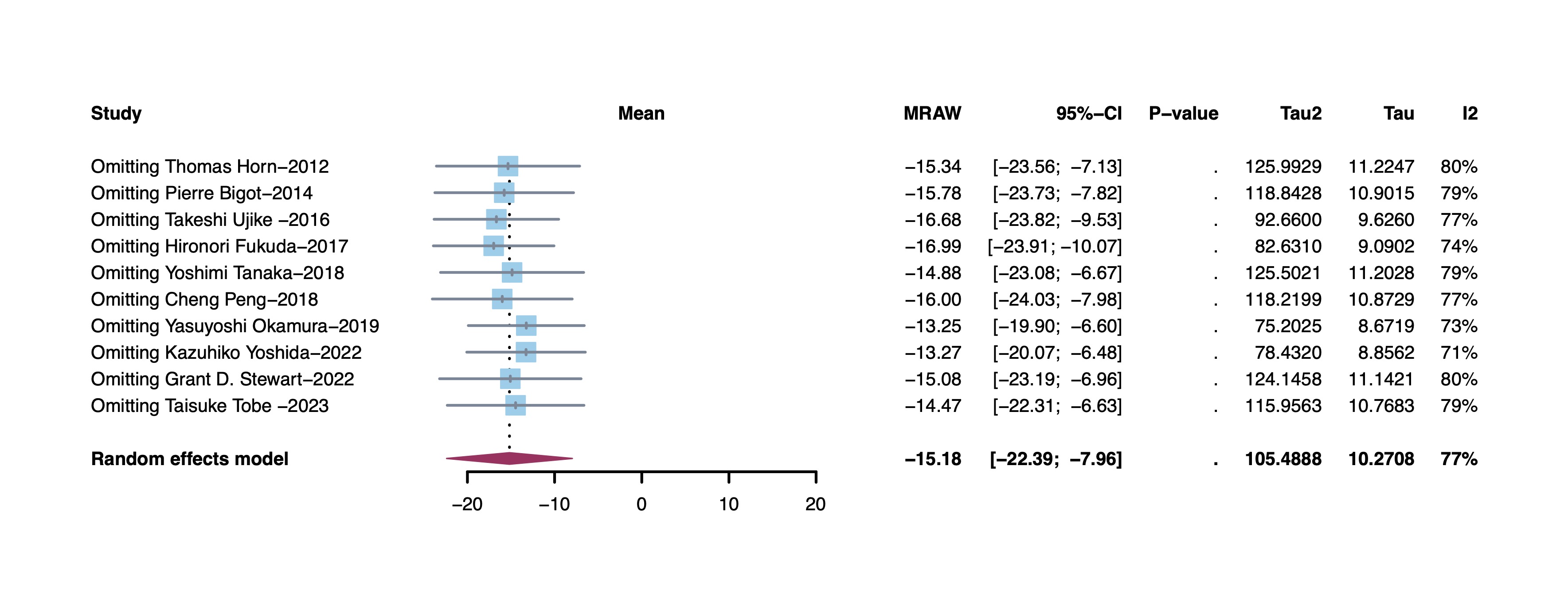

Supplement: Supplementary file 3 [file Image6.jpeg]

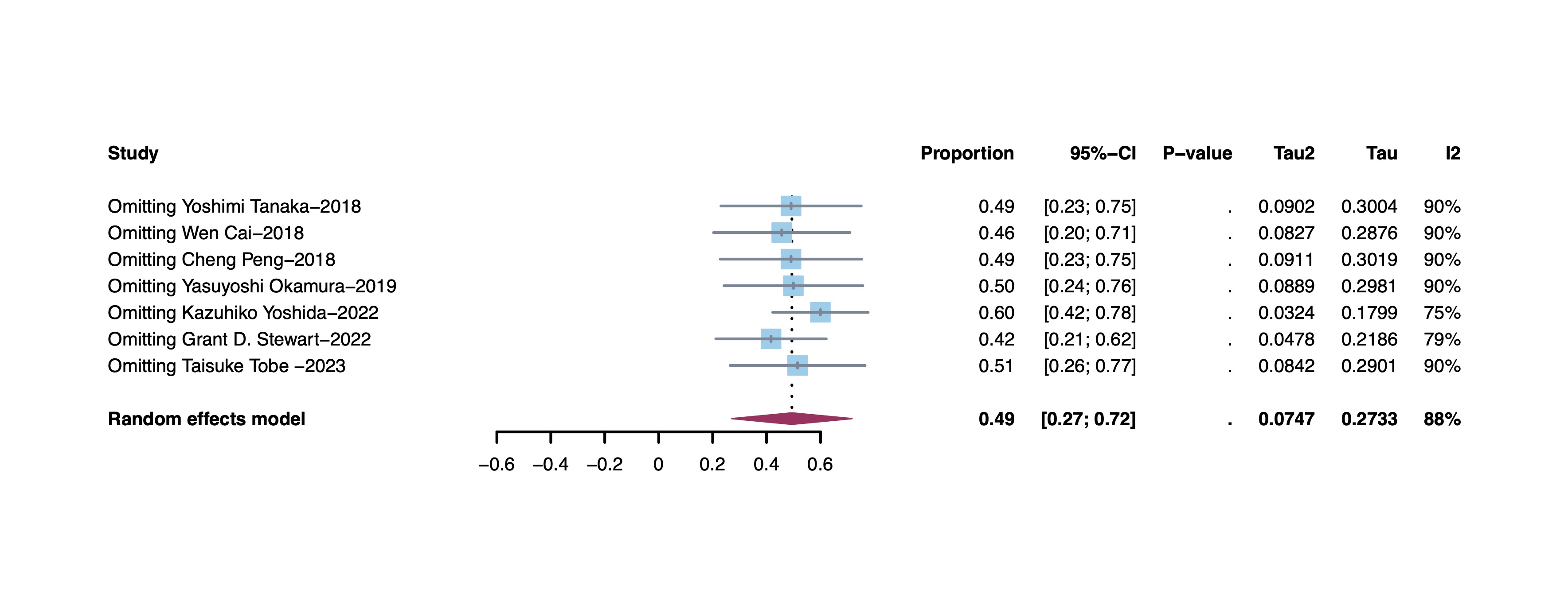

Supplement: Supplementary file 4 [file Image7.jpeg]

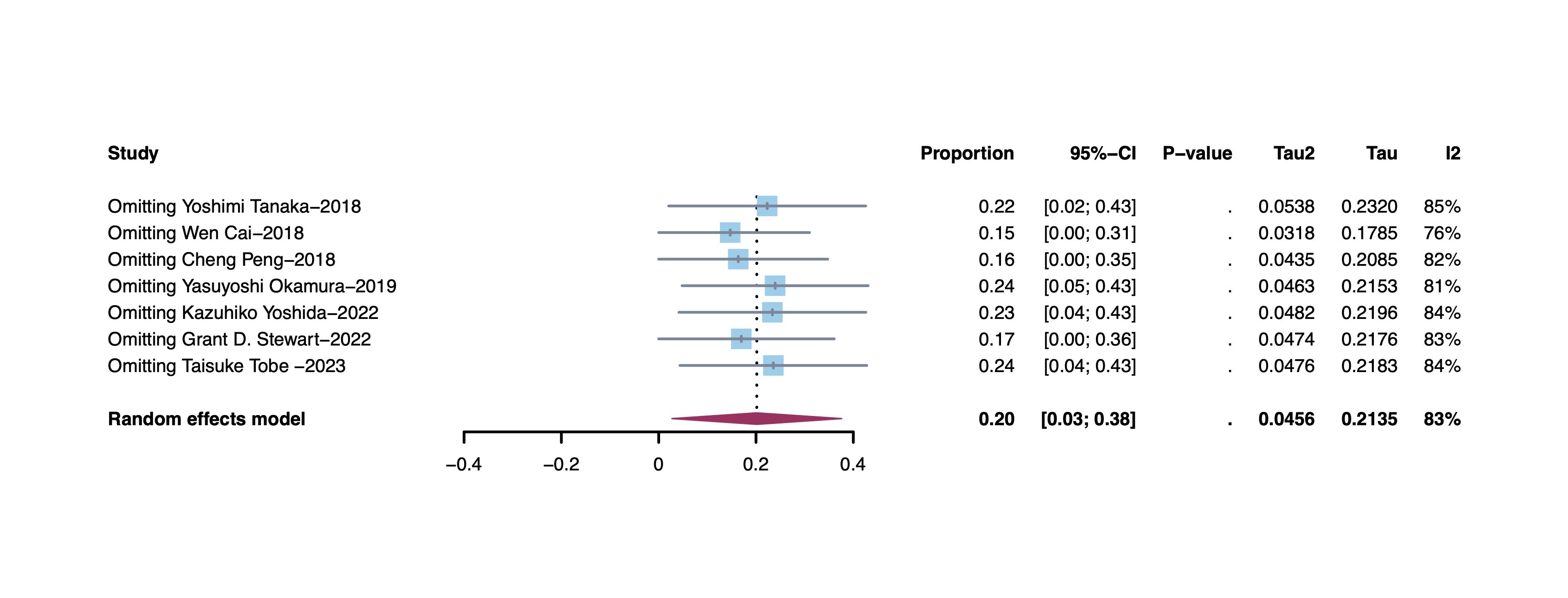

Supplement: Supplementary file 5 [file Image8.jpeg]

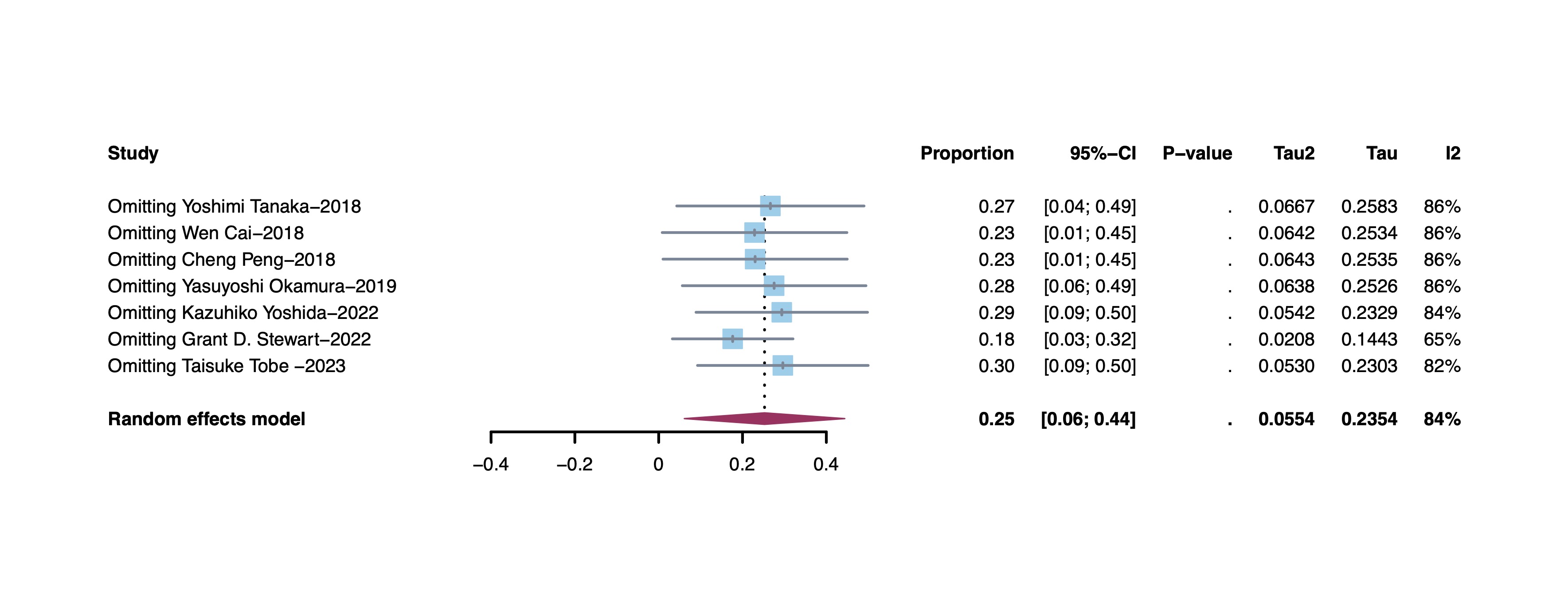

Supplement: Supplementary file 6 [file Image9.jpeg]

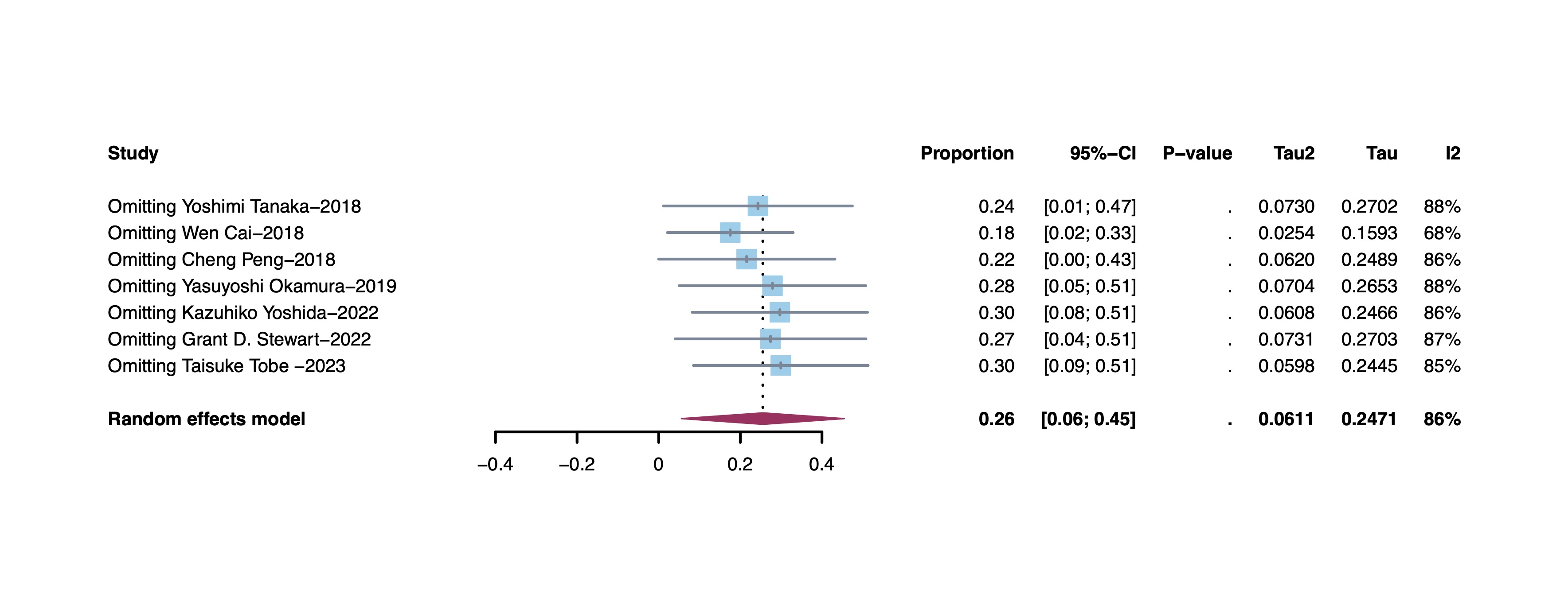

Supplement: Supplementary file 7 [file Image10.jpeg]

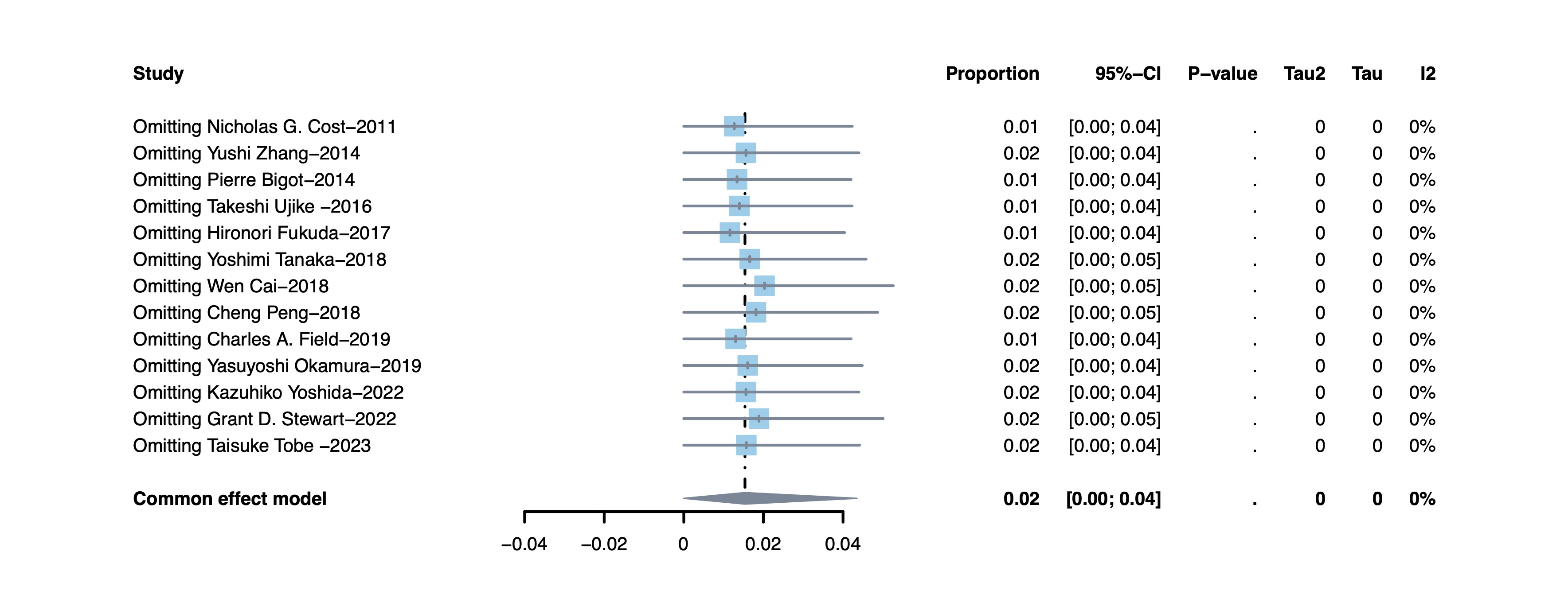

Supplement: Supplementary FIGURE 1 — Sensitivity analysis of reduced tumor thrombus levels. (A) Upstage Mayo grade. (B) Upstage Mayo grade 3/4. (C) Average Mayo grade changes. [file Image1.jpeg]

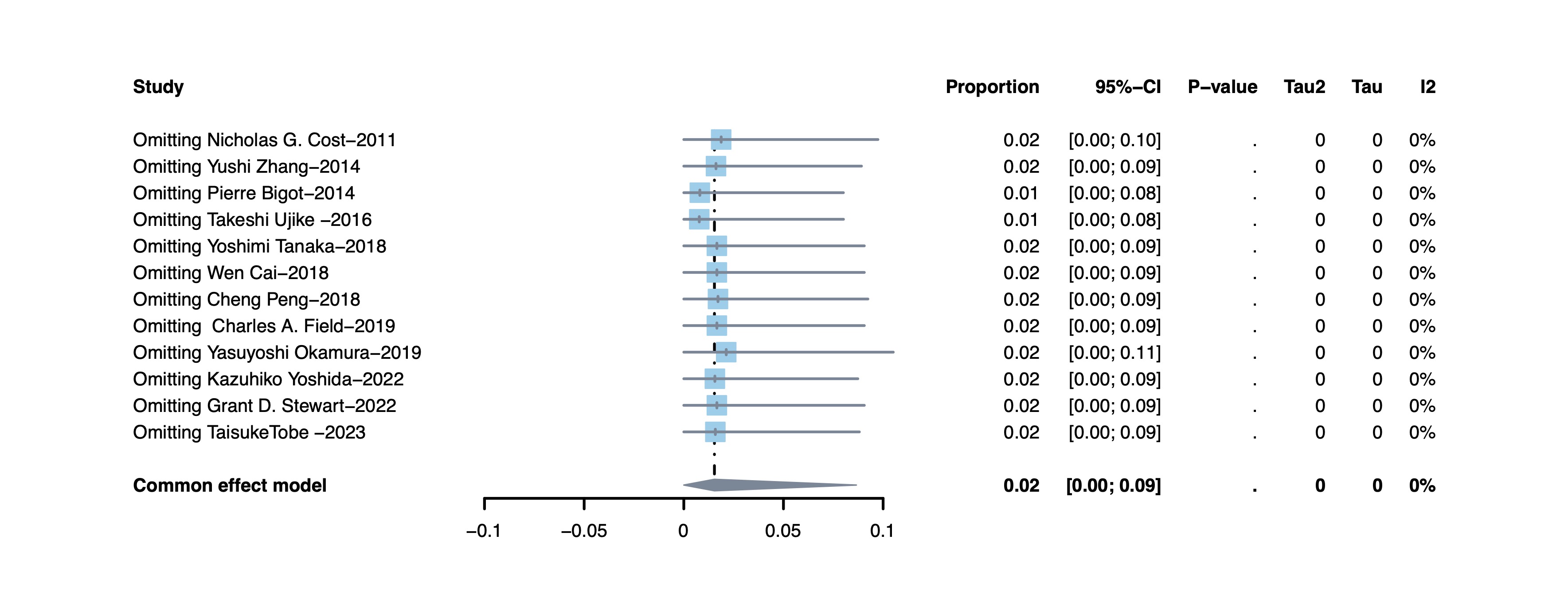

Supplement: Supplementary FIGURE 2 — Sensitivity analysis of changes of tumor thrombus. (A) Decrease in thrombus height. (B) Increase in thrombus height. (C) Average thrombus height changes. [file Image2.jpeg]

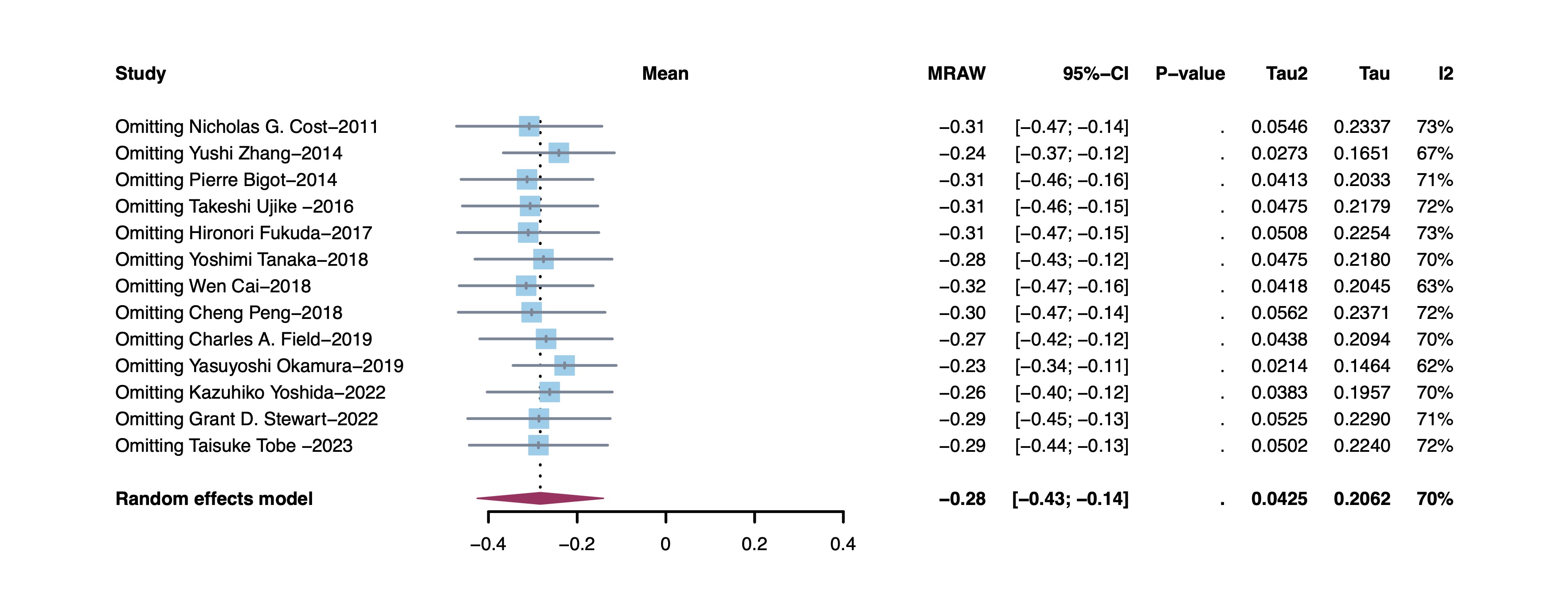

Supplement: Supplementary FIGURE 3 — Sensitivity analysis of adverse effects. (A) hypertension. (B) diarrhea. (C) fatigue. (D) hand-foot syndrome. [file Image3.jpeg]
